# Low Achieved Systolic Blood Pressure Related to Kidney Protection in Diabetic and Non-Diabetic High-Risk Hypertensive Patients

**DOI:** 10.1093/ajh/hpaf093

**Published:** 2025-05-21

**Authors:** Eirik Olsen, Camilla L Søraas, Roland E Schmieder, Kenneth Jamerson, Thomas M MacDonald, Giuseppe Mancia, Sondre Heimark, Maria H Mehlum, Knut Liestøl, Anne C K Larstorp, Julian E Mariampillai, Rune Mo, Lene V Halvorsen, Aud Høieggen, Morten Rostrup, Sverre E Kjeldsen, Michael A Weber

**Affiliations:** Norwegian University of Science and Technology, Trondheim, Norway; St. Olav`s University Hospital, Trondheim, Norway; Ullevaal University Hospital, Oslo, Norway; University of Oslo, Oslo, Norway; University of Erlangen, Erlangen, Germany; University of Michigan, Ann Arbor, Michigan, USA; University of Dundee, Dundee, Scotland, UK; University of Milan Bicocca, Milan, Italy; Ullevaal University Hospital, Oslo, Norway; Ullevaal University Hospital, Oslo, Norway; University of Oslo, Oslo, Norway; Ullevaal University Hospital, Oslo, Norway; University of Oslo, Oslo, Norway; Akershus University Hospital, Lørenskog, Norway; Norwegian University of Science and Technology, Trondheim, Norway; St. Olav`s University Hospital, Trondheim, Norway; Ullevaal University Hospital, Oslo, Norway; University of Oslo, Oslo, Norway; Ullevaal University Hospital, Oslo, Norway; University of Oslo, Oslo, Norway; Ullevaal University Hospital, Oslo, Norway; University of Oslo, Oslo, Norway; Ullevaal University Hospital, Oslo, Norway; University of Oslo, Oslo, Norway; University of Michigan, Ann Arbor, Michigan, USA; SUNY Downstate College of Medicine, New York, New York, USA

**Keywords:** blood pressure, creatinine, chronic kidney disease, end-stage kidney disease, hypertension, kidney function, the VALUE Trial

## Abstract

**BACKGROUND:**

Protecting the kidneys by lowering systolic blood pressure (SBP) in hypertensive patients is not unequivocally settled. We tested the hypothesis that achieving lower average SBP in middle-aged and older high-risk hypertensive patients with and without type-2 diabetes mellitus through several years would clarify kidney protection.

**METHODS:**

We analyzed patients 50–80 years with no cardiovascular events during the first 6 months of drug up-titration after randomization to valsartan or amlodipine, and with 3 or more visits onwards with standardized BP measurements. Adjusted Cox analyzes compared worsened kidney function defined as a 50% rise in se-creatinine on a minimum of two occasions at least 4 weeks apart or end-stage kidney disease (ESKD) in achieved SBP quartiles and in patients who achieved SBP < 130 and 130–139 mmHg with patients whose SBP remained ≥140 mmHg.

**RESULTS:**

A total of 13,803 patients were investigated of whom 4,655 had DM. Patients with DM had less worsened kidney function at SBP 130–139 mmHg (HR = 0.524, 95% CIs 0.375–0.733, *n* = 1849, *P* < 0.001) and at SBP < 130 mmHg (HR = 0.538, CIs 0.316-0.915, *n* = 674, *P* = 0.022) compared with patients at ≥ 140 mmHg. They also had less ESKD at SBP 130-139 mmHg (HR = 0.442, CIs 0.196–1.000, *P* = 0.050) with a similar trend at SBP < 130 mmHg and in quartile analysis with only 1 ESKD in the lowest quartile. Findings in patients without DM (*n* = 9,148) were similar to DM.

**CONCLUSIONS:**

In high-risk hypertensive patients aged 50–80 years, with and without DM, targeting SBP of 130–139 mmHg confers kidney protection with possible further benefit at the lower target of SBP < 130 mmHg.

**Clinical trials registration:**

Trial Number NCT06395194, www.clinicaltrials.gov.

## Graphical Abstract

**Graphical Abstract:**
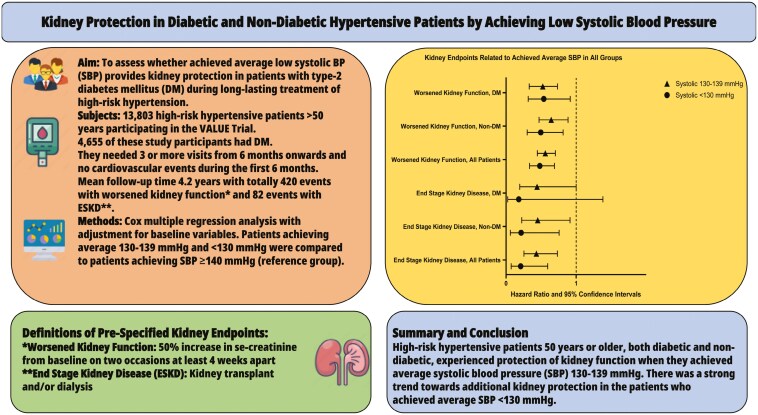

Hypertension and type-2 diabetes mellitus (DM) are closely related. Studies have shown that hypertension is twice as common among patients with DM as in the non-DM population.^1^ DM increases the burden of disease in hypertensive patients including cardiovascular death by 2-4-fold.^2,3^ Approximately 18–20% of the world’s population > 65 years is affected by DM, and 1/3 of these will develop chronic kidney disease (CKD).^4^ The combination of hypertension and DM significantly augments the risk of CKD^5^ whereas the presence of CKD increases the risk of hypertension, conversely worsening kidney function. The mechanism for the interplay between DM, hypertension and CKD is a complexity of factors beyond direct glomerular damage such as sodium retention, dyslipidemia, impaired microvascular function with pre-capillary re-modeling, endothelial dysfunction, large artery stiffness, sympathetic nervous system stimulation and activation of the renin-angiotensin-aldosterone system.^6^

Patients with DM and kidney involvement may have shortened life expectancy by as much as 16 years,^7^ making kidney protection a priority in DM. However few studies have investigated the optimal blood pressure (BP) to be achieved for protection against kidney disease in patients with DM. Though the mechanism(s) of kidney protection by achieving BP control is not known, it is likely explained by inhibiting or reversing one or more mechanisms in the complexity described above.^6^ Thus, a meta-analysis from 2013 found a kidney protective effect of intensive BP lowering in patients with CKD, but the study did not investigate DM patients.^8^ Current European Society of Hypertension (ESH) guidelines^9^ recommend targeting BP < 130/80 mmHg in DM patients < 65 years, and systolic BP (SBP) 130–139 mmHg in DM patients above 65 years.^9^ The American Diabetes Association (ADA)^3^ provides a Grade A recommendation for an on-treatment BP goal of <130/80 mmHg if it can be safely attained, mainly based on the Blood Pressure Control Target in Diabetes (BPROAD) study, which to date is the only prospective randomized controlled trial (RCT) showing less cardiovascular outcomes in favor of lower SBP targets (<120 vs. <140 mmHg) in a DM population.^3,10^ In the present study we therefore aimed to investigate whether, compared to patients remaining at SBP ≥ 140 mmHg, achieving average SBP 130-139 mmHg or SBP < 130 mmHg, will be related to preserved kidney function and prevention of end-stage kidney disease (ESKD) in middle-aged and older high-risk hypertensive patients with and without DM participating in the Valsartan Antihypertensive Long-term Use Evaluation (VALUE) Trial.

In a preliminary analysis^11^ we excluded patients with electrocardiographic left ventricular hypertrophy (ECG-LVH) because ECG-LVH may affect the relationships between achieved SBPs and outcomes.^12^ However, in the main analysis of kidney endpoints in VALUE^13^ we maintained the large group of *n* = 2458 patients with ECG-LVH when investigating kidney protection related to achieved average BP. Thus, with such precedence for investigating kidney outcomes related to achieved average SBPs with ECG-LVH patients included^13^ we have followed this approach also in the present study.

## METHODS

### Ethical considerations and data management

The VALUE trial was approved by ethics committees and written informed consent collected from all the participants in 31 countries. The data that support the findings of this study are available for analysis by the corresponding author upon reasonable request.

The VALUE Trial took place from 1997 to 2004. The sponsor was Novartis, who had an interactive role with the investigators for the study design, and Novartis monitored the 969 study sites and provided source data verifications. In 2011 Oslo University Hospital, Ullevaal, Norway, took over the database with conditions detailed in a written legal agreement with the previous funder. One author (S.E.K.) had from then full access to all the data in the study and took responsibility for its integrity and the data analysis. In 2016 one of the authors (M.H.M.) manually translated variables from the industrial SAS format to a SPSS format that could be used locally. Thus, the data file resides in the hands of the authors, and sponsors had no role in the present study.

### Study design

VALUE was a multicenter, prospective, double-blinded randomized clinical trial initiated and led by the investigators. The study compared the effects of two different antihypertensive treatment regimens on cardiac morbidity and mortality in hypertensive patients 50–80 years with 5% even older. One regimen was based on the angiotensin receptor blocker valsartan, and the other on the calcium channel blocker (CCB) amlodipine. At the study end the results showed similar numbers of the primary endpoint for both regimens.^14^

### Participants, study medications and follow-up

VALUE participants were selected based on predefined combinations of risk factors, including age above 50 years, male gender, smoking, total se-cholesterol > 6.2 mmol/L, and the presence of certain conditions such as ECG-LVH, determined by Cornell voltage-duration product or Sokolow–Lyon voltage criteria with or without a strain pattern, serum creatinine ≥ 150 and <265 μmol/l, proteinuria (positive urine dipstick at two occasions with a minimum of 4 weeks apart), DM, or verified coronary, cerebrovascular, or peripheral artery disease.^15^ The rationale for investigating hypertensive patients with high-risk like this, including 5% of patients being above the age of 80 years, was that despite all attempts to prevent study endpoints by optimal treatment of the high BP, endpoints are after all needed and thus we applied a risk based approach for hypertensive patients to qualify for participation.^15^

Diabetes mellitus was at the outset of the VALUE trial defined as the use of antidiabetic treatment or by the 1985 World Health Organization (WHO) criteria (fasting glucose 7.8 mmol/L [140 mg/dL] on two separate occasions). In 1999, during the course of the VALUE trial, WHO changed the definition of diabetes mellitus to a fasting blood glucose of 7.0 mmol/L (126 mg/dL) and/or blood glucose 11.1 mmol/L (200 mg/dL) 2 hours after oral intake of 75 g of glucose in venous plasma or serum (12.2 mmol/L [220 mg/dL] if capillary blood). During the blinded phase of the trial, the classification of new-onset diabetes was redefined to adhere to the WHO 1999 criteria, and this protocol change was prespecified in a study newsletter and later published.^16^

VALUE exclusion criteria^14^ included pregnancy, known renal artery stenosis, either myocardial infarction, percutaneous coronary intervention, or coronary artery bypass surgery during last three months, medically relevant valvular disease, cerebrovascular events last 3 months, severe hepatic disease, severe chronic kidney disease (serum creatinine ≥ 265 µmol/L, > 3.0 mg/dl), congestive heart failure requiring angiotensin converting enzyme inhibitor and patients using beta-blocker for both coronary artery disease and hypertension.

Patients who were previously treated for hypertension (92% of participants) were eligible if SBP was < 210 mmHg and DBP was < 115 mmHg. Untreated hypertensive patients were eligible if SBP was between 160 and 210 mmHg, and DBP was < 115 mmHg. Patients who already were on treatment, discontinued their previous antihypertensive medications when randomized to one of the trial’s masked study arms (valsartan or amlodipine) without a run-in phase (“rolled over”). Valsartan treatment started at a dosage of 80 mg daily, and amlodipine treatment started at 5 mg daily. If BP did not reach < 140/90 mmHg, the dose of either drug was doubled (160 mg or 10 mg, respectively), and hydrochlorothiazide (12.5 mg and 25 mg daily) and other antihypertensive drugs were added in sequential steps.

Participants were followed for 4–6 years to a maximum of 72 months, with monthly visits during the initial six months after randomization and later at six-month intervals. BP was measured at each visit using a calibrated sphygmomanometer or validated digital device with an appropriate size cuff as required by the protocol though the choice of measurement device was not harmonized between study centers because VALUE was not primarily a BP study but a hypertension outcome trial.

Patients were seated quietly for 5 min before the measurement of such standard research BP.^14,15^ BP was measured in the morning, 24 hours after the last dosing. Measurements were performed 3 times during each visit. Mean BP at each visit was defined as the average of all 3 readings. Monitoring was performed by the Novartis Medical Department with headquarters in Basel, Switzerland and East Hanover, NJ, USA, known for their strict monitoring routines including high quality-work with source data verification.

Blood samples were collected and analyzed at central labs located in Europe, North and South America, Israel, Australia, and South-Africa. Serum creatinine was measured at baseline and thereafter yearly.

### Kidney and cardiovascular outcomes

The primary goal of the study^14^ was to determine the time to the occurrence of the first cardiac event, which included a combination of fatal or non-fatal myocardial infarction, sudden cardiac death, death from revascularization procedures or heart failure, hospitalization for heart failure, and emergency procedures to prevent myocardial infarction. The secondary endpoints were all cardiovascular events, fatal and non-fatal stroke, myocardial infarction, hospitalized heart failure, cardiovascular-, non-cardiovascular-, and all-cause mortality. Pre-specified secondary kidney endpoints^13^ included worsened kidney function by at least a 50% increase in serum creatinine from baseline, captured at a minimum of two occasions with at least 4 weeks apart, and ESKD as need for dialysis and/or kidney transplantation. To ensure impartiality, the endpoint committee was not informed of the treatment assignments when assessing events. Thus, the current paper reports the protocol-defined pre-specified kidney endpoints, as they were reported by study investigators during the trial and approved by the VALUE Trial expert Endpoint Committee.^13,14^ However, since VALUE is the only mega-trial ever performed comparing head-to-head blocker of the renin-angiotensin system with amlodipine, explorative analyses of estimated glomerular filtration rate (eGFR) will likely be reported in the future. Regarding proteinuria, this was not quantified in VALUE and thus not reported.

### Statistical analysis

The VALUE Trial showed no difference between valsartan and amlodipine on the primary endpoint, and this allowed us to consolidate data from both treatment arms.^14^ Pooling of patients in the valsartan and amlodipine treatment arms was done for further analyses of the effects of BP lowering on the prevention of cardiovascular endpoints^17^ and this was further supported by no difference in the primary endpoint between valsartan and amlodipine for any pre-specified subgroup including the DM patients.^18^

**Figure 1.**
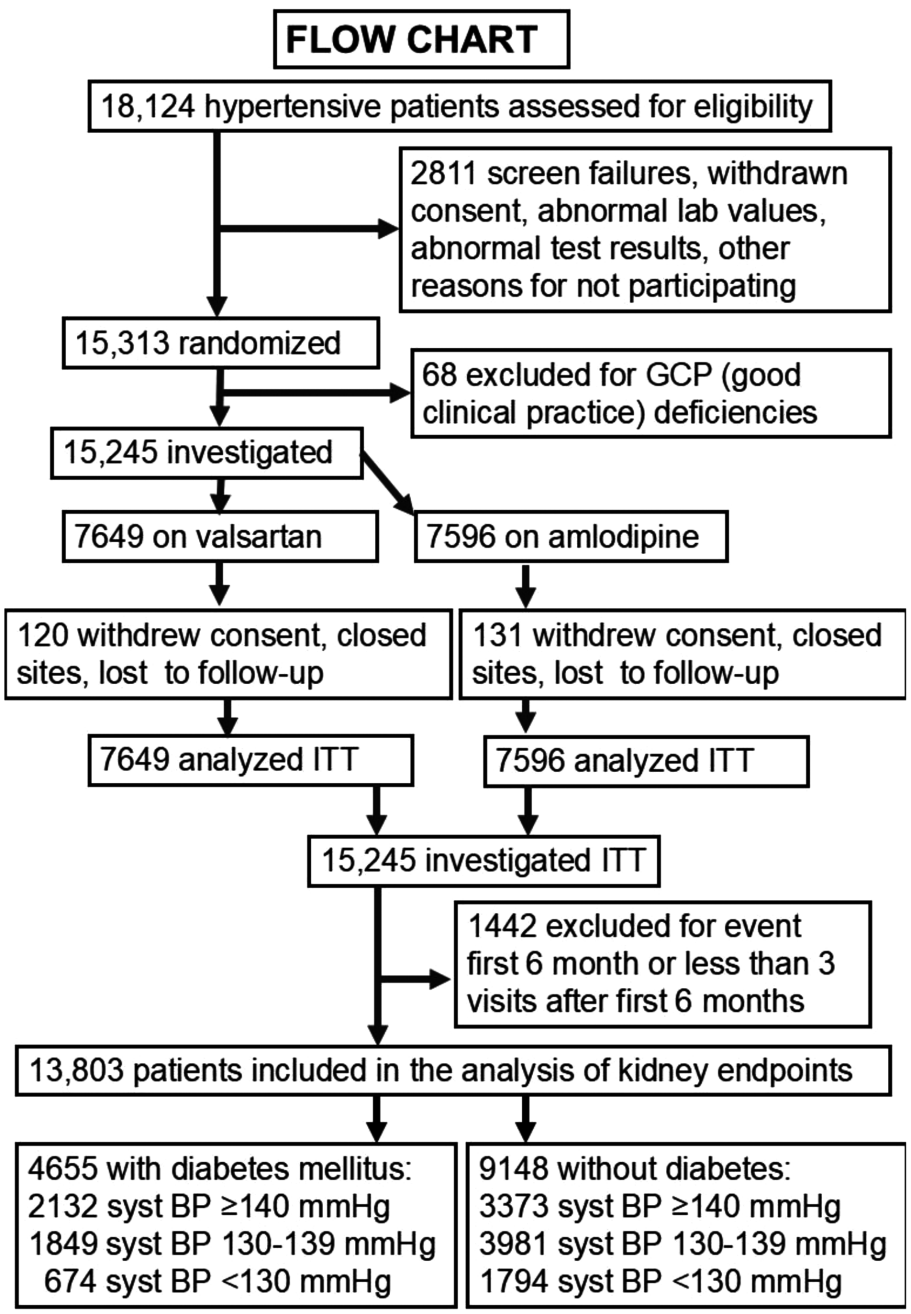
Flowchart showing that *n* = 15,245 patients with high-risk hypertension were investigated as intention to treat (ITT) for effects valsartan vs. amlodipine in the VALUE Trial. Further, *n* = 1442 patients were excluded from the current analysis either because they had endpoint during the first 6 months of up-titration of study drugs or they had less than 3 study visits with standardized office BP measurements after the first 6 months. A minimum of 3 visits was chosen a priori to be able to calculate an achieved average systolic BP during the trial. Then *n* = 13,803 patients were included in the analysis of incident kidney endpoints related to the achieved average systolic BP; *n* = 4,655 patients with diabetes mellitus and *n* = 9,148 patients without diabetes mellitus. These patients were then grouped according to their achieved average systolic BP ≥ 140 mmHg, 130-139 mmHg and < 130 mmHg.

Of the 15,245 hypertensive patients enrolled, 13,803 patients (**Figure 1**) did not experience any cardiovascular event during the initial six months of treatment and had attended a minimum of three study visits after that period. A minimum of three visits was decided a priori to ensure meaningful follow-up time.^19–21^ Patients with cardiovascular events during the first six months after randomization were excluded because of the uncontrolled and changing BP in the early phases of treatment with the randomized medications immediately following discontinuation of previous antihypertensive drugs; these early BPs were not representative for the later achieved average BPs.^22^ Statistical power was calculated for the original comparison of valsartan vs amlodipine for the primary endpoint but not any further for the present study.

The remaining 13,803 patients were analyzed, with 4,655 patients with DM at baseline and 9,148 patients in the non-DM group (**Figure 1**). Adjusted Cox proportional hazards models were used to analyze the endpoints for patients who achieved an average SBP of 130–139 mmHg or SBP < 130 mmHg up to the occurrence of a prespecified endpoint, or throughout the treatment period. The group with an achieved average SBP ≥ 140 mmHg was designated as the reference group. Further quartile analyses of achieved average SBP with the relationships to kidney endpoints were done by Cox regression with quartile one (highest SBP) as reference. Only a limited group of patients achieved average diastolic BP (DBP) ≥ 90 mmHg (*n* = 662) in the VALUE Trial and thus we do not report the kidney outcomes related to achieved average DBP.

The achieved average SBPs up to the occurrence of a prespecified event or throughout the trial duration, if no event occurred, were considered as the values that characterized the entire treatment period, except for SBPs during the first 6 months.^19–22^ Statistical significance was determined with a two-sided *P*-value of < 0.05 without adjustment for multiplicity. Data analysis was carried out using SPSS (IBM SPSS Statistics: Version: 28.0.1.0, Armonk, NY, USA). To minimize the influence of potential confounding factors, in all analyses of SBP ranges, SBP quartiles and trends, hazard ratios (HRs) were adjusted for treatment allocation and for baseline covariates including, age, sex, SBP, DBP, body mass index, high total se-cholesterol (> 6 mmol/L), smoking status, proteinuria, ECG-LVH, previous myocardial infarction, previous stroke and peripheral artery disease (**Table 1**). Trend analyses were performed by using SBP ranges or quartiles coded as continuous variables in Cox regression analyses. Data are presented as means with standard deviations (SDs), absolute numbers with percentages in parentheses, or point estimates with 95% confidence intervals (CIs).

**Table 1.** Baseline variables for type 2 diabetes mellitus (DM) and non-DM related to ranges of achieved SBP.

Variables	All DM (*n* = 4,655)	All non-DM (*n* = 9,148)	DM ≥140 mmHg (*n* = 2,132)	Non-DM ≥140 mmHg (*n* = 3,373)	DM 130–139 mmHg (*n* = 1,849)	Non-DM 130–139 mmHg (*n* = 3,981)	DM <130 mmHg (*n* = 674)	Non-DM <130 mmHg (*n* = 1,794)
Female sex	2051 (44.1%)	3813 (41.7%)	1017 (47.7%)	1554 (46.1%)	785 (42.5%)	1649 (41.1%)	249 (36.9%)	610 (34.0%)
Age (years)	67.0 (7.9)	67.1 (8.2)	67.9 (7.6)	68.6 (8.1)	66.5 (7.8)	67.0 (7.9)	65.4 (8.3)	64.8 (8.2)
BMI (kg/m^2^)	29.9 (5.3)	28.0 (4.7)	30.0 (5.1)	28.0 (4.7)	29.8 (5.4)	28.0 (4.6)	30.1 (5.8)	27.8 (4.8)
Treatment amlodipine	2364 (50.8%)	4567 (49.9%)	1008 (47.3%)	1555 (46.1%)	993 (53.7%)	2110 (53.0%)	363 (53.9%)	902 (50.3%)
Treatment valsartan	2291 (49.2%)	4581 (50.1%)	1124 (52.7%)	1818 (53.9%)	856 (46.3%)	1871 (47.0%)	311 (46.1%)	892 (49.7%)
No.visits after 6 m	8.5 (1.8)	8.8 (1.6)	8.5 (1.9)	8.6 (1.8)	8.7 (1.7)	8.9 (1.5)	8.5 (1.8)	8.8 (1.6)
European origin	4014 (86.2%)	8350 (91.3%)	1913 (89.7%)	3163 (93.8%)	1567 (84.7%)	3638 (91.4%)	534 (79.2%)	1549 (86%)
African origin	265 (5.7%)	277 (3.0%)	119 (5.6%)	97 (2.9%)	90 (4.9%)	107 (2.7%)	56 (8.3%)	73 (4.1%)
Asian origin	181 (3.9%)	314 (3.4%)	36 (1.7%)	53 (1.6%)	106 (5.7%)	162 (4.1%)	39 (5.8%)	99 (5.5%)
Other ethnicity	195 (4.2%)	207 (2.3%)	64 (3.0%)	60 (1.8%)	86 (4.7%)	74 (1.9%)	45 (6.7%)	73 (4.1%)
SBP (mmHg)	156.0 (18.8)	153.9 (19.0)	163.4 (18.1)	162.0 (18.6)	152.2 (16.3)	151.8 (16.6)	142.8 (17.5)	142.9 (17.8)
DBP (mmHg)	87.0 (10.7)	87.9 (10.8)	87.7 (10.9)	88.6 (11.2)	87.0 (10.2)	87.9 (10.3)	84.6 (11.3)	86.6 (10.9)
HR (beats/min)	74.2 (10.7)	71.3 (10.5)	74.4 (10.8)	71.5 (10.8)	74.2 (10.7)	71.2 (10.5)	73.6 (10.5)	71.0 (10.1)
Creatinine (µmol/l)	99.7 (23.6)	100.8 (22.8)	100.9 (24.7)	102.1 (24.7)	98.5 (22.7)	100.0 (22.1)	99.3 (22.3)	100.3 (20.1)
Atrial fibrillation	131 (2.8%)	201 (2.2%)	64 (3.0%)	75 (2.2%)	57 (3.1%)	85 (2.1%)	10 (1.5%)	41 (2.3%)
Smoking (yes)	949 (20.4%)	2379 (26.0%)	361 (16.9%)	806 (23.9%)	427 (23.1%)	1059 (26.6%)	161 (23.9%)	514 (28.7%)
History of MI	1833 (39.4%)	4468 (48.8%)	733 (34.4%)	1440 (42.7%)	742 (40.1%)	1914 (48.1%)	358 (53.1%)	1114 (62%)
History of PAD	561 (12.1%)	1337 (14.6%)	284 (13.3%)	540 (16.0%)	216 (11.7%)	581 (14.6%)	61 (9.1%)	216 (12.0%)
History of stroke	729 (15.7%)	1970 (21.5%)	321 (15.1%)	754 (22.4%)	295 (16.0%)	835 (21.0%)	113 (16.8%)	381 (21.2%)
Proteinuria	1299 (27.9%)	1766 (19.3%)	661 (31.0%)	678 (20.1%)	465 (25.1%)	729 (18.3%)	173 (25.7%)	359 (20.0%)
High cholesterol	1344 (28.9%)	3301 (36.1%)	673 (31.6%)	1386 (41.1%)	504 (27.3%)	1439 (36.1%)	167 (24.8%)	476 (26.5%)

Numbers represent mean (SD) for continuous variables and the absolute number with percentages in parentheses for categorical variables. In all analyses of SBP ranges, SBP quartiles and trends, hazard ratios were adjusted for treatment allocation and for the baseline covariates age, sex, SBP, DBP, body mass index (BMI), high se-cholesterol (> 6.2 mmol/L), smoking habits, proteinuria, ECG-LVH, previous myocardial infarction (MI), previous stroke/transient ischemic attack (TIA) and known peripheral artery disease (PAD). HR; heart rate m; months.

## RESULTS

### Patients’ characteristics

**Figure 2.**
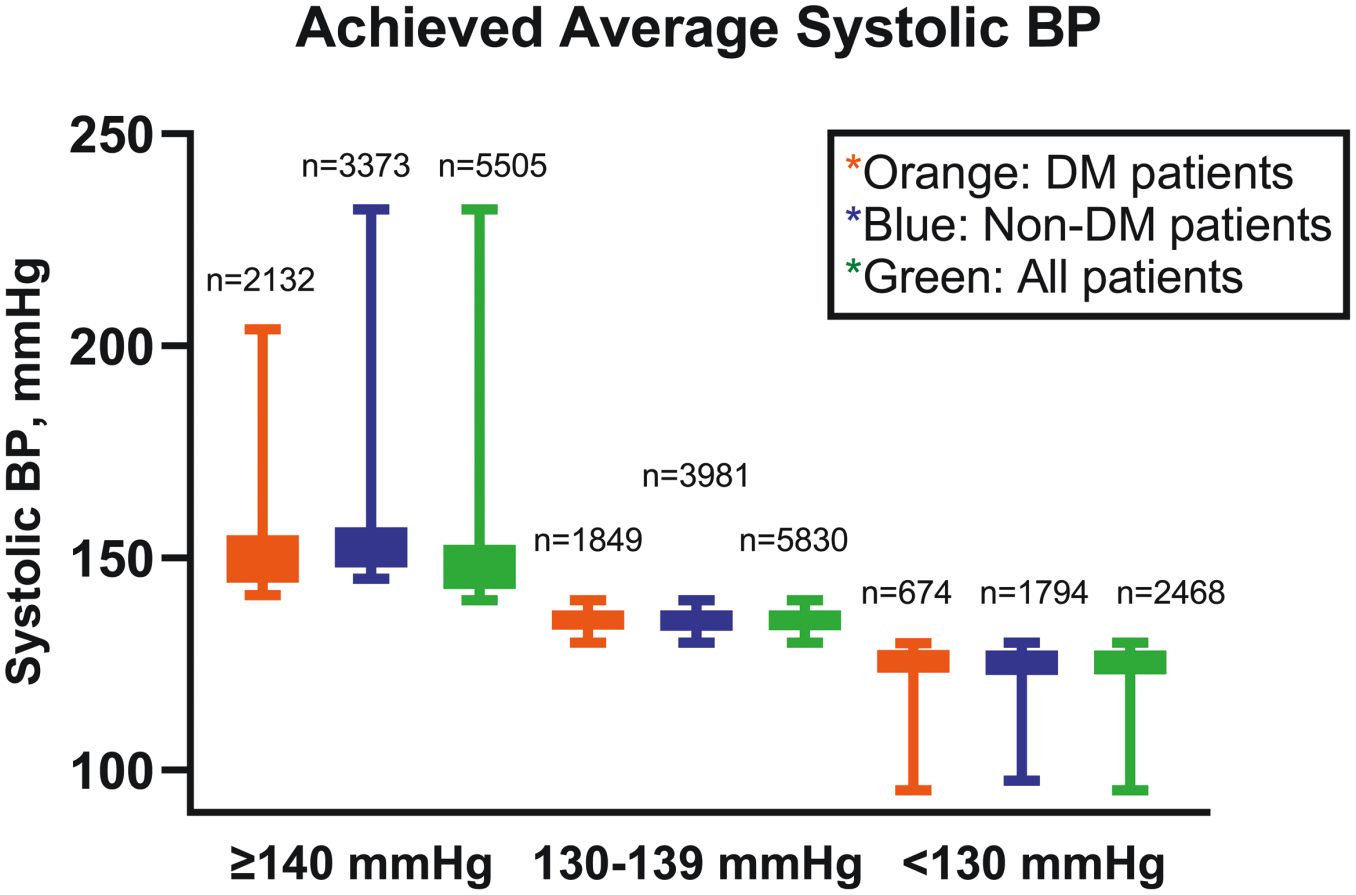
The Box plots show achieved average SBP in patients with diabetes (DM, *n*=4,655), non-DM (*n*=9,148) and in the total group (*n*=13,803) for ranges of achieved average SBP from 6 months and throughout. The patients were grouped according to their achieved average systolic BP ≥140 mmHg (mean values 150, 149 and 149 mmHg in the 3 groups, respectively), 130-139 mmHg (mean values 135 mmHg in all 3 groups) and <130 mmHg (125 mmHg in all 3 groups). Patients with DM are shown in orange Box plots, patients without DM in blue Box plots, and all patients are shown in green Box plots. Patients with achieved average systolic SP 130-139 mmHg have a minimal scatter as expected. The group who achieved average systolic BP ≥140 mmHg has a marked scatter towards high values for the 4^th^ quartile of the distribution, whereas the group who achieved average systolic BP <130 mmHg has a marked scatter towards low values for the 1^st^ quartile of the distribution. The patients were followed by 969 investigators in 31 countries in all parts of the world. Target systolic BP was <140 mmHg which was achieved by 70% of the patients. The main driver for the achieved systolic BP in individual patients, and thus in the groups, was the investigators’ ability to follow the study protocol and up-titrate study medication. This investigator inertia is explained in the Discussion.

The average age of the DM Group (*n* = 4,655) was 67 years with 44 % females and 86 % of European origin. The non-DM Group (*n* = 9,148) averaged 67 years, with 42 % females, and 91 % were of European origin. Differences between the DM and the non-DM Group at baseline were higher average BMI (30 ± 5 vs 28 ± 5 kg/m^2^) and higher SBP (156 ± 19 vs 154 ± 19 mmHg) in the DM Group. The non-DM Group had higher numbers of daily smokers, history of myocardial infarction, known peripheral artery disease and previous stroke/transient ischemic attack than the DM Group. When stratifying the DM Group in ranges of achieved average SBP (*n* = 2,132 with ≥ 140 mmHg, *n* = 1,849 within 130-139 mmHg, *n* = 674 with < 130 mmHg) the group achieving average < 130 mmHg, at baseline had lower age, fewer patients of European origin, lower SBP and DBP, more smokers, and more previous myocardial infarction than the other groups. Achieved average SBPs in the ranges of SBP in the groups were comparable (**Figure 2**; **Table 1**).

### Kidney endpoints

**Figure 3.**
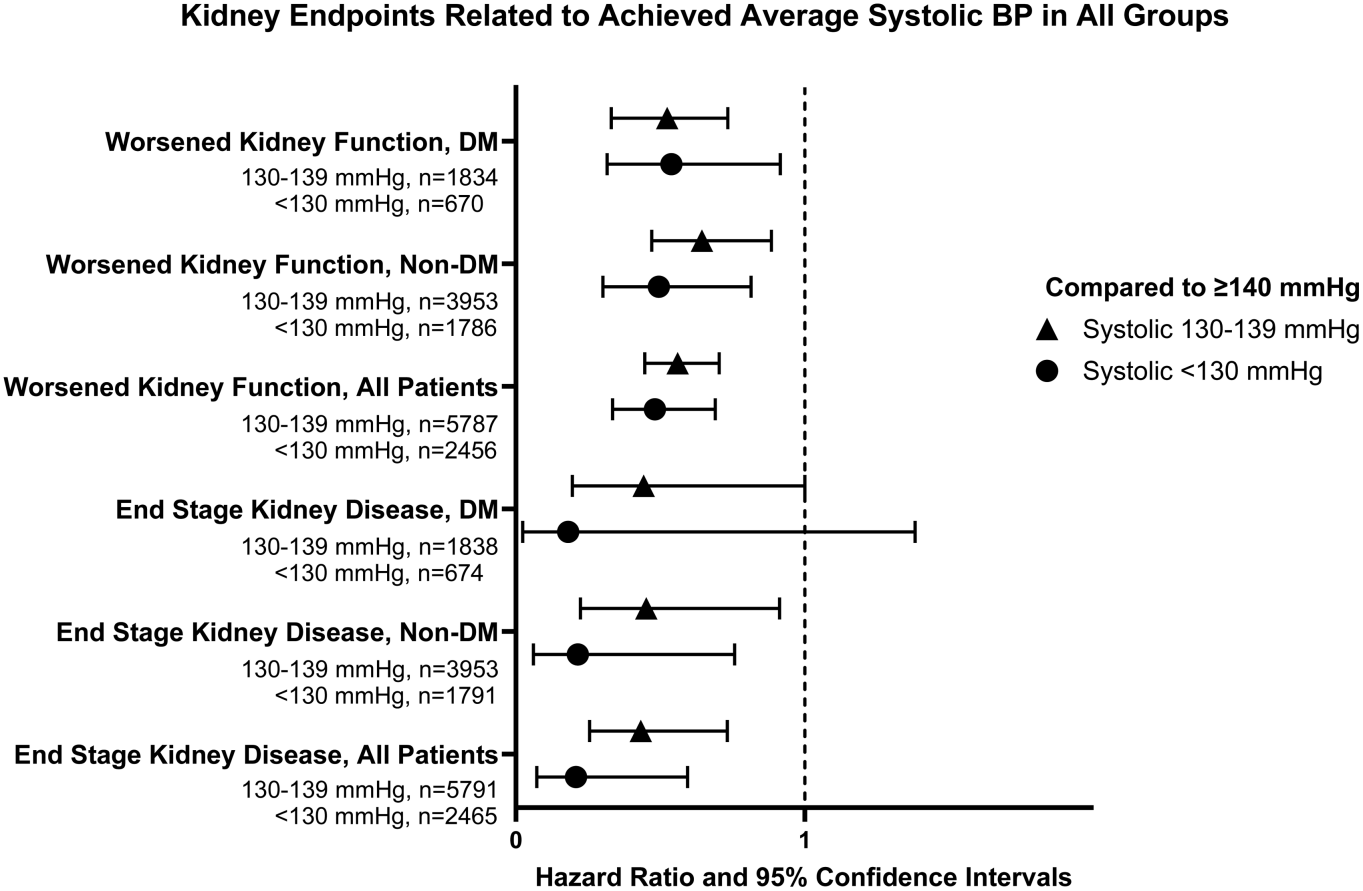
The Forest plots show Hazard ratios with 95% confidence intervals for the study protocol-defined kidney endpoints approved by the VALUE Trial endpoint committee during the trial. The kidney endpoints are presented related to ranges of achieved average systolic BP, 130–139 mmHg and < 130 mmHg, compared to ≥ 140 mmHg (defined as 1.0) in patients with diabetes (DM, *n* = 4,655), patients without (non) DM (*n* = 9,148) and in the total group of all patients (*n* = 13,803). The endpoint Worsened Kidney Function, detected in *n* = 420 patients, was defined as a 50% increase of serum creatinine at two occasions with a minimum of 4 weeks apart. The endpoint End Stage Kidney Disease (ESKD), detected in *n* = 82 patients, was defined as the need for dialysis or transplantation. Statistical analyses were done with adjusted Cox proportional hazards models. ESKD was stepwise lower with lower systolic BP for all groups though borderline significant (*P* = 0.05) for DM patients achieving an average of 130-139 mmHg and not significant for DM patients achieving average systolic BP < 130 mmHg (only one DM patient had ESKD at achieved average systolic BP < 130 mmHg). *The number of patients with kidney endpoints (n and %) in the groups is shown in*Table 2.

**Figure 4:**
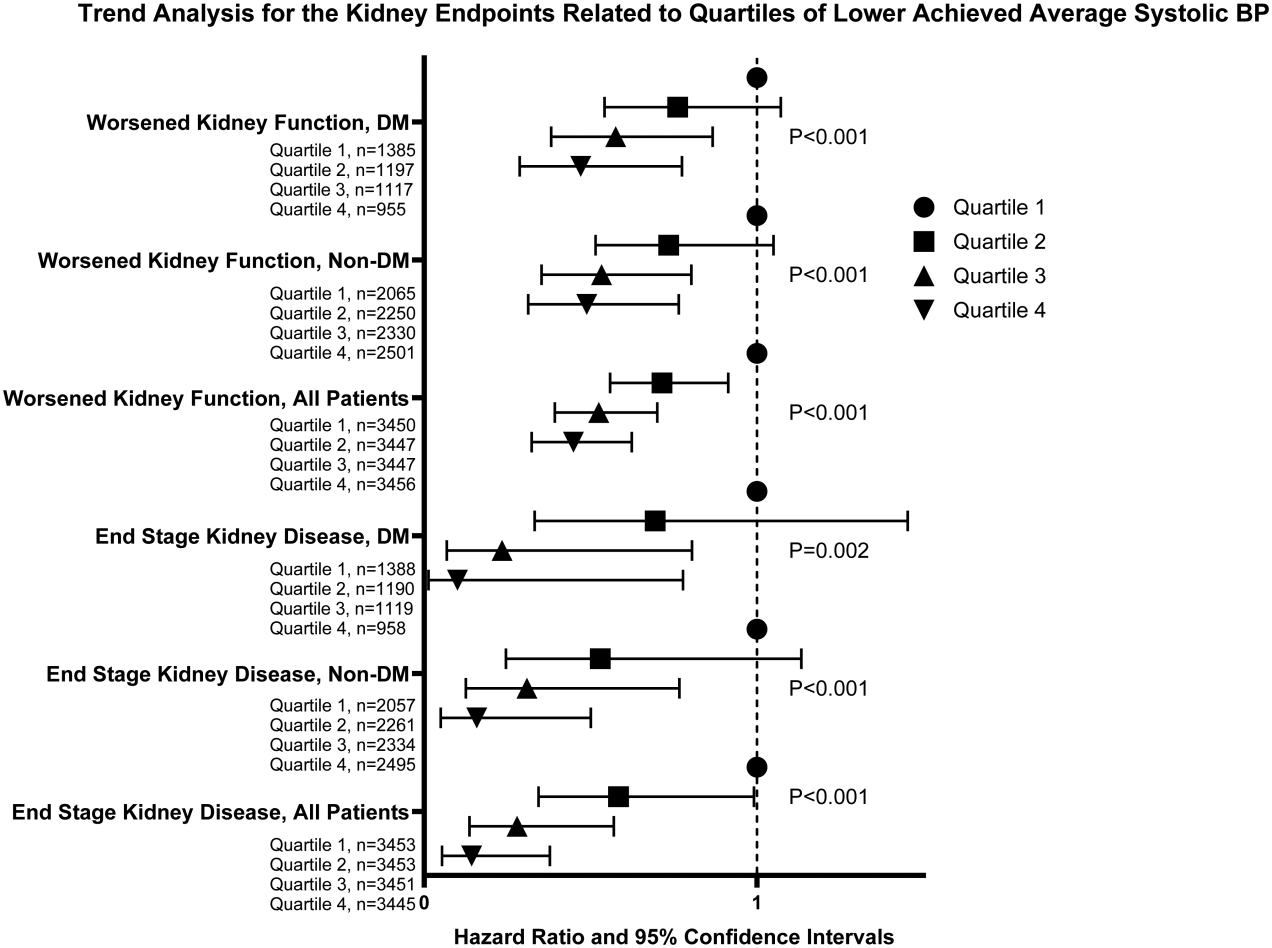
The Forest plots show Hazard ratios with 95% confidence intervals for the study protocol-defined kidney endpoints approved by the VALUE Trial endpoint committee during the trial. The kidney endpoints, defined in legends to Figure 3, are presented related to quartiles of achieved average systolic BP with quartile 1 defined as 1.0. The Forest plots show the main findings from the continuous quartile trend analyses in patients with diabetes (DM, *n* = 4,655), non-DM (*n* = 9,148) and in the total group (*n* = 13,803). Statistical analyses were done with adjusted Cox proportional hazards models. The trend for less kidney endpoints with lower quartiles of achieved systolic BP is statistically highly significant for the analyses of both kidney endpoints (Worsened Kidney Function and ESKD) in patients with DM, in patients without (non-) DM and in the group of all patients. *The number of patients with kidney endpoints (n and %) in the groups is shown in*Table 3.

In the present analysis of the VALUE Trial *n* = 420 patients had worsened kidney function and *n* = 82 patients had ESKD as approved pre-specified secondary endpoint. The analyses of these kidney endpoints are presented in Figures 3 and 4 and in Tables 2 and 3.

**Table 2.** Kidney endpoints in the DM group (*n* = 4,655), in the total group (*n* = 13,803) and in the non-DM group (*n* = 9,148) related to ranges of achieved average SBP (mmHg).

Groups and endpoints	SBP ≥ 140 mmHg (reference)	SBP 130–139 mmHg	SBP < 130 mmHg
**DM group** Worsened kidney function	*n* = 148/2150 (6.9%)	*n* = 51/1834 (2.8%) HR 0.524 (CIs 0.375–0.733) *P* < 0.001	*n* = 17/670 (2.5%) HR = 0.538 (CIs 0.316–0.915) *P* = 0.022
**DM group** End-stage kidney disease (ESKD)	*n* = 32/2143 (1.5%)	*n* = 8/1838 (0.4%) HR = 0.442 (CIs 0.196–1.000) *P* = 0.050	*n* = 1/674 (0.1%) HR = 0.181 (CIs 0.024–1.381) *P* = 0.099
**Total group** Worsened Kidney Function	*n* = 262/5557 (4.7%)	*n* = 120/5787 (2.1%)HR = 0.560 (CIs 0.446–0.704), *P* < 0.001	*n* = 38/2456 (1.5%),HR = 0.481 (CIs 0.335–0.690), *P* < 0.001
**Total group** End-stage kidney disease (ESKD)	*n* = 57/5546 (1.0%)	*n* = 21/5791 (0.4%),HR 0.432 (CIs 0.255–0.732), *P* = 0.002	*n* = 4/2465 (0.2%),HR = 0.208 (CIs 0.073–0.594), *P* = 0.003
**Non–DM group** Worsened kidney function	*n* = 114/3407 (3.3%)	*n* = 69/3953 (1.7%) HR = 0.644 (CIs 0.470–0.884), *P* = 0.006	*n* = 21/1786 (1.2%) HR = 0.495 (CIs 0.301–0.814), *P* = 0.006
**Non-DM group** End-stage kidney disease (ESKD)	*n* = 25/3403 (0.7%)	*n* = 13/3953 (0.3%) HR = 0.451 (CIs 0.224–0.912) *P* = 0.027	*n* = 3/1791 (0.2%) HR = 0.215 (CIs 0.061–0.757) *P* = 0.017

*N* denotes number of events in each SBP range divided by number of patients. Percent (%) events in brackets.

Adjusted Cox proportional hazards models were used to analyze the endpoints for patients who achieved average SBP of 130-139 mmHg or SBP < 130 mmHg up to the occurrence of a prespecified endpoint, or throughout the treatment period. Hazard ratios (HRs) with 95% confidence intervals (CIs) were adjusted for treatment allocation and for baseline covariates including, age, sex, SBP, DBP, body mass index, high total se-cholesterol (> 6.2 mmol/L), smoking status, proteinuria, ECG-LVH, previous myocardial infarction, previous stroke and known peripheral artery disease.

**Table 3.** Kidney endpoints in the DM group (*n* = 4,655), in the total group (*n* = 13,803), and in the non-DM Group (*n* = 9,148) related to quartiles of achieved average SBP (mmHg)

Groups and endpoints	Quartile 1 (reference)	Quartile 2	Quartile 3	Quartile 4
**DM group** Worsened kidney function	SBP = 154.5 (9.3) *n* = 106/1385 (7.7%)	SBP = 141.9 (1.9) *n* = 54/1197 (4.5%) HR = 0.762 (CIs 0.542–1.072)*P* = 0.117	SBP = 135.1 (1.6) *n* = 34/1117 (3.0%) HR = 0.576 (CIs 0.382–0.867)*P* = 0.008	SBP = 126.8 (4.7) *n* = 22/955 (2.3%) HR = 0.471 (CIs 0.287–0.775) *P* = 0.003
**DM group** End stage kidney disease (ESKD)	SBP = 154.3 (9.3) *n* = 26/1388 (1.9%)	SBP = 141.0 (1.9) *n* = 11/1190 (0.9%) HR = 0.694 (CIs 0.332–1.453) *P* = 0.333	SBP = 135.1 (1.6) *n* = 3/1119 (0.3%) HR = 0.234 (CIs 0.068–0.805)*P* = 0.021	SBP = 126.8 (4.7) *n* = 1/958 (0.1%) HR = 0.100 (CIs 0.013–0.778)*P* = 0.028
**Total group** Worsened kidney function	SBP = 153.8 (9.1) *n* = 186/3450 (5.4%)	SBP = 140.8 (1.9) *n* = 108/3447 (3.13%) HR = 0.715 (CIs 0.559–0.914) *P* = 0.007	SBP = 135.1 (1.6) *n* = 71/3447 (2.1%) HR = 0.525 (CIs 0.393–0.701) *P* < 0.001	SBP = 126.6 (4.8) *n* = 55/3456 (1.6%) HR = 0.449 (CIs 0.323–0.624) P < 0.001
**Total group** End stage renal disease (ESKD)	SBP = 153.7 (9.0) *n* = 45/3445 (1.3%)	SBP = 140.8 (1.9) *n* = 22/3451 (0.6%) HR = 0.584 (CIs 0.344–0.991) *P* = 0.046	SBP = 135.1 (1.6) *n* = 10/3453 (0.3%) HR = 0.279 (CIs 0.136–0.570) *P* < 0.001	SBP = 126.6 (4.8) *n* = 5/3453 (0.1%) HR = 0.143 (CIs 0.054–0.378) P < 0.001
**Non–DM group** Worsened kidney function	SBP = 153.3 (8.8) *n* = 80/2065 (3.9%)	SBP = 140.7 (1.9) *n* = 54/2250 (2.4%) HR = 0.735 (CIs 0.515–1.050)*P* = 0.091	SBP = 135.1 (1.6) *n* = 37/2330 (1.6%) HR = 0.533 (CIs 0.353–0.803)*P* = 0.003	SBP = 126.5 (4.8) *n* = 33/2501 (1.3%) HR = 0.489 (CIs 0.313–0.765) *P* = 0.002
**Non–DM group** End stage renal disease (ESKD)	SBP = 153.2 (8.8) *n* = 19/2057 (0.9%)	SBP = 140.7 (1.9) *n* = 11/2261 (0.5%) HR = 0.529 (CIs 0.246–1.134)*P* = 0.102	SBP = 135.1 (1.6) *n* = 7/2334 (0.3%) HR = 0.309 (CIs 0.125–0.767)*P* = 0.011	SBP = 126.5 (4.8) *n* = 4/2495 (0.2%) HR = 0.158 (CIs 0.050–0.501)*P* = 0.002

*N* denotes number of events in each quartile divided by number of patients. Percent (%) events in brackets.

Results are reported as mean (SD) and hazard ratio (HR) with 95% confidence intervals (CI) adjusted for baseline variables. Adjusted Cox proportional hazards models were used to analyze the endpoints for patients who achieved average SBP in the quartiles up to the occurrence of a prespecified endpoint, or throughout the treatment period. Hazard ratios (HRs) with 95% confidence intervals (CIs) were adjusted for treatment allocation and for the baseline covariates age, sex, SBP, DBP, body mass index, high total se-cholesterol (> 6.2 mmol/L), smoking status, proteinuria, ECG-LVH, previous myocardial infarction, previous stroke and known peripheral artery disease.

### Kidney endpoints in the DM Group (*n* = 4,655) related to ranges of achieved average SBP

DM patients achieving average SBP 130–139 mmHg (HR = 0.524, 95% CIs 0.375–0.733, *P* < 0.001) and SBP < 130 mmHg (HR = 0.538, 95% CIs 0.316–0.915, *P* = 0.022) had fewer worsened kidney function endpoints. The ESKD endpoint was lower in the DM Group achieving average SBP 130–139 mmHg (HR = 0.442, 95% CIs 0.196–1.000, *P* = 0.050), whereas ESKD was not significantly reduced at SBP < 130 mmHg with only one event (HR = 0.181, 95% CIs 0.024–1.381, *P* = 0.099) (**Figure 3 and Table 2** ).

### Kidney endpoints in the DM Group (*n* = 4,655) related to quartiles of achieved average SBP

The quartile analysis of kidney endpoints in relation to achieved average SBP confirmed the results of the above-reported SBP range analysis. The most striking findings were the lower worsened kidney function endpoint both in quartile 3 (*P* = 0.008) and in quartile 4 (*P* = 0.003) and the lower ESKD in the same quartiles, *P* = 0.021 and *P* = 0.028, respectively, though there were only three patients with this event in quartile 3 and only one patient in quartile 4. However, few patients with ESKD in quartiles 3 and 4 suit well as a sign of nephroprotection by low achieved SBP. Continuous trend analysis (**Figure 4**) was highly significant (*P* < 0.001) for the worsened kidney function endpoint (i.e., lower endpoint incidence with the lowest achieved SBP). The trend analysis was also significant for the ESKD endpoint (*P* = 0.002) (**Figure 4**, **Table 3**).

### Kidney endpoints in the non-DM group (*n* = 9,148)

Achieved average SBP 130–139 mmHg was significantly associated with less of the worsened kidney function endpoint (HR = 0.644, CIs 0.470–0.884, *n* = 3953, *P* = 0.006), with a further lower worsened kidney function with SBP < 130 mmHg (HR = 0.495, CIs 0.301–0.814, *n* = 1786, *P* = 0.006) in the non-DM Group (**Table 2,Figure 3**). The ESKD endpoint was also statistically lower in the 130–139 mmHg (HR = 0.451, CIs 0.224–0.912, *P* = 0.027) and < 130 mmHg (HR = 0.215, 95% CIs 0.061–0.757, *P* = 0.017) SBP ranges, with, however, only 3 patients with ESKD < 130 mmHg in the latter group (**Figure 3**). In the quartile analysis of achieved average SBP, the worsened kidney function endpoint was lower in quartiles 3 (*P* = 0.003) and 4 (*P* = 0.002) in the non-DM Group (**Table 3**) and the ESKD endpoint significantly lower in quartiles 3 (*P* = 0.011) and 4 (*P* = 0.002). Continuous trend analysis (**Figure 4**) was highly significant (*P* < 0.001) for both the worsened kidney function and for the ESKD endpoint in non-DM.

### Kidney endpoints in the total group (*n* = 13,803)

In the total group, achieved average SBP 130–139 mmHg was significantly associated with fewer worsened kidney function endpoints (HR = 0.560, 95% CIs 0.446–0.704, *n* = 5787, *P* < 0.001), and with a further lower worsened kidney function in the group achieving SBP < 130 mmHg (HR = 0.481, 95% CIs 0.335-0.690, *n* = 2456, *P* < 0.001) (**Figure 3**). In this group the ESKD endpoint was lower in the SBP range 130–139 mmHg (HR = 0.432, 95% CIs 0.255–0.732, *P* = 0.002) and <130 mmHg (HR = 0.208, 95% CIs 0.073–0.594, *P* = 0.003) (**Figure 3**). Continuous trend analyses (**Figure 4**) showed *P* < 0.001 for both less worsened kidney function and ESKD for lower achieved SBP.

## DISCUSSION

In middle-aged and older DM patients with high-risk hypertension of the VALUE trial we found that an achieved average SBP in the range of 130–139 mmHg was associated with fewer patients with worsened kidney function and ESKD than those seen in patients remaining with an on-treatment SBP ≥ 140 mmHg. In addition, we found that there was a significantly reduced hazard ratio of worsened kidney function in patients who achieved average SBP < 130 mmHg. ESKD was also lower at achieved SBP < 130 mmHg, though at SBP < 130 mmHg there were few patients with the ESKD endpoint and thus statistically a less pronounced improvement of kidney outcome. In non-DM patients, kidney endpoint reductions with achieved average SBP 130–139 mmHg and <130 mmHg were almost identical to findings in the DM Group. This was the case also for the pooled DM and non-DM groups as well as for continuous and quartile-based analyses of achieved SBP. Taken together these data suggest that an achieved average SBP of <130 mmHg is associated with kidney protection in high-risk hypertensive patients with and without DM.

The clinical potency of valsartan up against amlodipine in previously treated high-risk hypertensive patients was not known at the outset of VALUE in 1997.^23^ The difference in achieved BP (lower on amlodipine) may have influenced the main outcome of the Value Trial. However, the primary endpoint was almost identical in the two study arms suggesting that potential amlodipine benefits in lowering BP may have been out-balanced by valsartan’s angiotensin-II inhibiting properties.^14^ Examples of such properties, as previously published from the Value Trial, are less incident atrial fibrillation on valsartan^24^ and less incident diabetes mellitus.^16^

As we recently summarized^13^ there is a strong relationship between high BP and the risk of developing CKD and in people with CKD, a high BP is associated with a faster decline of kidney function towards ESKD. However, randomization to intensive systolic BP lowering did not slow the progression of CKD in the Modification of Diet in Renal Disease (MDRD)^25–27^ or in the African American Study of Kidney Disease and Hypertension (AASK).^28,29^ Though, long-term follow-up in MDRD showed significantly (32%) reduced risk of ESKD in the treatment arm with the lowest BP.^27^ In AASK, there was an increased risk of combined CKD progression and ESKD with intensive BP control. Summary evidence, however, shows that BP treatment slows the progression of CKD.^13^ In hypertension without CKD, a meta-analysis of ten RCTs, with altogether 317 kidney endpoints, found no reduction in kidney outcomes in patients randomized to antihypertensive treatment or an intensive BP lowering strategy.^30^ However, in the Losartan Intervention For Endpoint Reduction in Hypertension study (LIFE) patients achieving SBP ≤ 130 mmHg had a slower decline in estimated glomerular filtration rate (eGFR).^31^ In the Avoiding Cardiovascular Events through Combination Therapy in Patients Living with Systolic Hypertension study (ACCOMPLISH), treatment target for patients who were included with CKD was SBP < 130 mmHg and the benazepril plus amlodipine combination provided a better kidney protection than benazepril plus hydrochlorothiazide.^32^ A better kidney protection in ACCOMPLISH has now been shown to be related also to a better cardiovascular protection.^33^

The Antihypertensive and Lipid-Lowering Treatment to Prevent Heart Attack Trial (ALLHAT) was the largest double-blinded RCT and randomized high-risk hypertensive patients to target BP < 140/90 mmHg on chlorthalidone (*n* = 15,255) compared to doxazocin (*n* = 9061), amlodipine (*n* = 9048) or lisinopril (*n* = 9054).^34,35^ The rate of ESKD was the same on all 4 drugs and fairly like the rates of ESKD we reported in VALUE.^13^ More recently the ALLHAT investigators^36^ found that the ESKD rate at the 2-year visit in some patients with apparent treatment resistant hypertension (*n* = 1870 with uncontrolled BP on 3 or more drugs) was higher than in patients with BP control on 3 medications (*n* = 12,814, HR = 1.95, 95% CIs = 1.11–3.41). These data suggest that in ALLHAT patients with more severe hypertension had higher rates of ESKD in support of our current findings in VALUE.

The Action to Control Cardiovascular Risk in Diabetes (ACCORD) study^37^ did not show the benefit of lowering SBP < 120 mmHg compared to < 140 mmHg except for the reduction of stroke as a secondary endpoint. Worsened kidney function was not a prespecified endpoint in ACCORD, but clinical measures revealed significantly more patients in the intensive therapy group with increased serum creatinine, glomerular filtration rate (GFR) < 30 ml/min/1.73m^2^ and kidney failure, than in the standard therapy group. ACCORD had a younger population and stricter BP control than VALUE. However, the benefits of BP lowering in ACCORD were diluted by the triple factorial design^38–40^ and the negative effects of intensive glycemia treatment. Also, the target SBP of < 120 mmHg in ACCORD may have been too low. The SBP range of 120–130 mmHg could be optimal for kidney protection in patients with DM and hypertension.

A subgroup analysis of the SPRINT study^41^ investigated effect on intensive SBP lowering on kidney and cardiovascular outcomes in a group of patients with GFR > 60mL/min/1.73m^2^. The study found transient rises in serum creatinine and concluded that these effects were outweighed by benefits in all-cause death and cardiovascular events.^42^ SPRINT did not include patients with DM, but our current analyses of patients without DM suggest a beneficial outcome on CKD with lower SBP also in the non-DM group. However, direct comparisons of BPs between SPRINT and VALUE is complicated by the heterogeneous approach of SPRINT to BP measurements (attended and unattended BP).^43^

In the HOPE study^44^ comprising patients with high normal BP or already treated hypertension (average on-treatment BP of 139/75 mmHg), BP lowering treatment provided significant reduction of all-cause mortality, stroke, myocardial infarction, and cardiovascular death compared to placebo, even though the patients only experienced a modest reduction in BP. A sub-study of HOPE investigating the DM patients (*n* = 3,577) found an even greater risk reduction of the main composite cardiovascular endpoint, and importantly a 24% reduction in overt nephropathy.^45^

A strength of our VALUE sub-study is the high number of DM patients (*n* = 4,655) included. Furthermore, the endpoints were prespecified in the main study protocol, assessed by an expert endpoint committee with nephrology leadership, and the number of events was high, mainly due to a high-risk population, long follow-up time and strictly standardized procedures and measurements in the VALUE trial with few (*n* = 90) patients lost to follow-up.^14^ Also, we found the worsened kidney function endpoint (50% increase of serum creatinine) consistent through Cox regression analyses assessing BP groups, BP quartiles, and among non-DM patients.

Amlodipine and valsartan have differential effects on renal function. Hemodynamic effects of valsartan and amlodipine as first line treatments may directly influence glomerular filtration rate made clearly visible in a specific head-to-head study.^46^ RAS-blockers may lead to a rise in se-creatinine through dilatation of afferent and efferent arterioles and reduced filtration pressure in the glomeruli, while CCB may influence serum creatinine by dilatation of afferent arterioles only, leading to glomerular hyperfiltration.^46^ It is also well-known that amlodipine and RAS-blocker may act differently on urinary protein excretion. However, these differential drug effects on renal function did not influence the risk of ESKD in DM vs. non-DM patients in the VALUE Trial.

Our findings provide support for lowering SBP to 130–139 mmHg in middle-aged and older high-risk DM and non-DM hypertensive patients, as this reduces the risk of prespecified cardiovascular and kidney endpoints. These findings are in line with the findings of a systematic review.^47^ With achieved average SBP < 130 mmHg we found significant reductions in the risk of worsened kidney function and support for reduced incidence of ESKD. Thus, SBP < 130 mmHg may provide renal protection, which is an important endpoint in patients with DM, though this low SBP was neutral regarding cardiovascular endpoints and mortality in the same patients. A complicating issue is that patients with ECG-LVH who achieved average SBP < 130 mmHg may increase cardiac and all-cause mortality^12^ but preserve kidney function.^31^

Until recently there existed no evidence from clinical studies that SBP should aim at < 120 mmHg to prevent endpoints in patients with hypertension and DM.^48^ However, with the publication of BPROAD^10^ there is support for target SBP < 120 mmHg in hypertensive patients with DM to prevent cardiovascular endpoints. However, BPROAD^10^ did not have a risk high enough and/or did not last long enough to provide kidney endpoints. Besides, the target SBP in between 140 and 120 mmHg, namely < 130 mmHg, is still unresolved by BPROAD data.^10^

The ongoing OPTIMAL-DIABETES study is interesting and hopefully, it will show enough kidney endpoints for the investigators to make statistical assessments (https://clinicaltrials.gov/study/NCT04040634?term=optimal%20diabetes&rank=4). However, when reviewing the design of the OPTIMAL-DIABETES study we see that target SBP below 140, 130 or 120 mmHg for cardiovascular endpoints will likely not be resolved in as much as the study is comparing < 140 vs. < 120 mmHg.

Further, it is interesting to discuss stages of hypertension and risk level as indications for drug treatment. In the VALUE Trial all patients had established hypertension with excessive additional risk variables and without doubt clear indication for optimal antihypertensive drug treatment according to updated ADA^3^ and ESH^9^ guidelines. However, in patients without established hypertension (below 140 mmHg in seated office BP) we are not in support of risk-based treatment of hypertension—a topic some of us have discussed in recent commentary papers.^49,50^

In a previous analysis of the relations between achieved BP and cardiovascular outcomes in VALUE, using different analytical methods,^51^ reducing BP consistently to <140/90 mmHg had marked beneficial effects both when data were calculated as proportion of visits at BP target or as on-treatment mean BP. Reducing BP to <130/80 mmHg led to a possible further benefit on stroke prevention, whereas the risk of other outcomes remained similar to, or slightly greater than that seen at the higher target. When extensively adjusting all analyses for covariates including the pre-specified inclusion criteria,^52^ on the other hand we could not detect signs of any J-curve or rise in risk of cardiovascular endpoints at the lowest achieved BPs.^52^ The mathematical nadirs for the optimal BPs to prevent cardiovascular endpoints in VALUE were then 128/75 mmHg.^52^ However, the kidney endpoints were not included in previous analyses.^51,52^ When more specifically analyzing the cardiovascular outcomes in the study participants with DM, and with the ECG-LVH patients included, the overall picture was that the cardiovascular endpoints in VALUE were prevented at achieved BP < 140/90 mmHg but less <130/80 mmHg whether patients had DM or belonged to the general high-risk hypertensive population.^53,54^

Some VALUE patients (*n* = 1298) developed DM during the trial.^16^ We have not done subgroup-analysis of these patients, likely to have higher risk of kidney endpoints than patients with never-DM. We may speculate whether it could give the non-DM group a lower incidence of kidney events if these patients who developed DM during the trial were excluded from the non-DM group.

It could be questioned whether the decrease in BP in the VALUE Trial was due to better kidney function, or vice versa, and postulated that the cause-consequence relationship cannot be determined. A mega-trial like VALUE was not organized primarily to investigate detailed mechanisms of BP reduction but to investigate treatment prevention of endpoints. However, interesting findings could be extracted from monitoring data during the blinded phase of a large outcome RCT.^55^ In general, SBP control, usually aiming at a target <140 mmHg in hypertension comparative RCTs, was importantly much better than in clinical practice but far from perfect as visualized by the group of patients remaining ≥140 mmHg in **Figure 2**. Approximately 50% of patients in LIFE, 60% in the Anglo Scandinavian Cardiac Outcomes Trial (ASCOT), 70% in VALUE, and more than 80% in ACCOMPLISH achieved the target systolic BP given in the protocol. But in all these major comparative outcome trials the achieved average BP was not determined by patient-specific factors but by the investigators’ ability to up-titrate study medication according to the protocol. In all these RCTs even with extensive and detailed monitoring and source data verification, what we characterized as “investigator inertia” was the main driver of achieved BP in the individual patients. The reasons why study investigators had such a reluctant attitude towards up-titrations of medication to achieve BP control are unknown though we have speculated extensively in our report.^55^ As always in outcome mega-trials the cause-consequence relationship cannot always be determined with 100% certainty though the outcomes that we report in VALUE were mainly due to the decreases in BP in the study in as much as achieved BP in individual patients was determined by investigator’s adherence with the up-titration scheme in the study protocol.

The first 6 months after randomization were dedicated to up-titration of medication and gaining BP control in the VALUE Trial. We have previously in detail reported data related to this phase of the trial and follow-up in the subsequent 2 years.^22^ For example, at 6 months 62% of study patients had combined systolic and diastolic BP control < 140/90 mmHg. From this time point and onwards BP remained rather stable with a limited gain in BP control slowly rising to 70% for SBP < 140 mmHg towards the end of the study.

VALUE is 20 years old^14^; in current times patients also take other ARBs,^56,57^ statins,^58,59^ finerenone,^60,61^ sodium-glucose-cotransporter-2 inhibitors,^62–64^ or glucagon-like peptide-1-receptor agonists,^65^ which can improve cardiovascular and kidney outcomes of patients with DM and/or kidney disease. On the other side, for similar reasons, the type of head-to-head comparison of antihypertensive drug regimens such as ARB vs CCB, like in VALUE, can unlikely any longer be done and the now frequent co-administration of the other nephroprotective drugs may lead to even larger difficulties with investigating kidney endpoints in a hypertensive population, rendering the present VALUE results important.

### Study limitations

Even if we consider the present results regarding SBP as consistent and strong, our analyses have limitations. Whilst BPROAD^10^ and ACCORD^37,38^ were prospective RCTs with different target BPs (SBP < 120 vs < 140 mmHg), the present study provides an observational aspect of a prospective, double-blinded RCT which compared outcomes of two different drug regimens. Observational data are mainly hypothesis-generating. Therefore, to reduce the risk of incidental findings, we analyzed the kidney endpoints also for non-DM patients, and stratified the patients into quartiles, adjusted Cox hazard models for treatment allocations and numerous baseline characteristics and found the results consistent through all performed analyses.

Kidney function as se-creatinine or eGFR has been reported in many outcome studies with varying results though mostly in line with the changes on the RAS-blocker valsartan and the CCB amlodipine as observed in VALUE.^13^ However, it is a general limitation that ESKD to our knowledge has been previously reported only in a limited number of hypertension outcome trials.^34,35,43^

The statistical power analysis in VALUE was related to the primary aim namely comparison of two drug regimens.^14^ As visible in the Results section, some of the kidney endpoint analyses were underpowered including assessments of the ESKD endpoint in the group with DM. Further, all participants were survivors of the first 6 months, and the patients who were investigated had to survive three later study visits which may have introduced a limited but unavoidable immortal time bias.

## Conclusions

We found that patients with DM but also patients without DM who achieved average SBP 130–139 mmHg had less worsened kidney function and ESKD compared to patients who remained with achieved average SBP ≥ 140 mmHg. Patients who achieved average SBP < 130 mmHg also had a marked reduction of the kidney endpoints though the number of patients with ESKD at achieved SBP < 130 mmHg was too low to allow a strong conclusion regarding this most serious endpoint for this achieved SBP target level. However, our findings were consistent in both DM and non-DM patients. Though cardiovascular prevention is optimal for achieved SBP 130-139 mmHg in the VALUE Trial patients with DM,^11,13^ our data suggest that achieved SBP < 130 mmHg possibly provides further kidney protection.

Our findings should ideally be supported by randomized data before being recommended in clinical practice. However, CKD is a serious consequence of DM and hypertension in combination. Beyond any doubt patients who achieved average SBP < 130 mmHg in our study preserved kidney function. Based on our kidney observations, also in light of cardiovascular prevention at low achieved average SBP in the recently published BPROAD study^10^ we do not hesitate to recommend SBP < 130 mmHg as achieved average treatment target in high-risk middle-aged and older hypertensive patients if treatment is well tolerated. Preserved kidney function would normally translate into less ESKD: We observed a strong trend for this to take place, though we conclude more carefully for ESKD whether our hypertensive patients had DM or not.
